# Towards an understanding of the ethics of electronic consent in clinical trials

**DOI:** 10.1186/s13063-024-08330-3

**Published:** 2024-08-16

**Authors:** Katherine Sahan, Rohan Wijesurendra, David Preiss, Marion Mafham, Mark Sheehan

**Affiliations:** 1 Clinical Trial Service Unit, Oxford Population Health, University of Oxford, Richard Doll Building, Old Road Campus, Roosevelt Drive, Oxford, OX3 7LF UK; 2 Ethox Centre, Oxford Population Health, University of Oxford, Big Data Institute, Old Road Campus, Roosevelt Drive, Oxford, OX3 7LF UK

## Abstract

There are good practical reasons to use electronic consent (e-consent) in randomised trials, especially when conducting large-scale clinical trials to answer population-level health research questions. However, determining ethical reasons for e-consent is not so clear and depends on a proper understanding of what e-consent means when used in clinical trials and its ethical significance. Here we focus on four features of ethical significance which give rise to a range of ethical considerations relating to e-consent and merit further focused ethics research.

## Main text

### Practical reasons for development of e-consent

Informed consent is a fundamental legal and ethical requirement in research involving human participants, including clinical trials for investigational medical products (CTIMPs). It is the instantiation of the ethical requirement that participants actively choose and so, provide autonomous authorisation to take part in the study [1]. It ensures that participants are able to exercise their autonomy free from coercion and with sufficient information and comprehension of what participation means [2]. Conventionally, obtaining informed consent would involve provision of written paper information and an in-person, face-to-face discussion between participant and researcher before providing a wet ink signature on a consent form. Importantly, various aspects of these conventions have been challenged on practical grounds across a range of contexts.

First, the need for paper has been questioned. Paper forms can be long, legalistic, and hard to comprehend. Using paper-based processes may not suit some research contexts [3] and may give rise to logistical and security challenges (e.g. where, how to store paper records) [4]. Second, the need for in-person interaction or discussion during the consent process has been queried for certain types of research, such as research where participating involves only some or no clinic visits and therefore meeting in-person may be burdensome or extraneous for both participants and researchers. These research studies are referred to as remote, home-based [5, 6],^1^ direct-to-consumer or direct-to-participant studies. More recently such studies have included formal CTIMPs [8, 9].

Large-scale CTIMPs to answer population health research questions present challenges that are paradigmatic of the practical challenges of conventional consent. When recruiting thousands of participants, it may simply be unfeasible (for example, costly or inefficient) to consent each patient in-person and obtain their consent documented on paper. Nor may any clinic visits be necessary, as study drugs can be mailed to the home (ASCEND PLUS,^2^ LENS,^3^ [9]) or even administered within the home where this involves professionals having to infuse drugs [10]. This has led to the development of alternatives to conventional consent processes. These can include remote consent where paper forms (invitation, screening, information, and consent) are mailed out to participant’s homes [9, 11] without the need for an in-person interaction. The remote consent process can also be run electronically.^4^ Paper forms can be sent in electronic copy by email, or the information can be presented using web-based platforms which create a ‘user profile’ for the participant (or provide an individualised link) and take her through a set of web pages and forms, sometimes using visual information such as animated videos instead of written text. The consent form itself can be an electronic form, signed using a secure system and logged in the trial’s electronic storage. Again, an in-person interaction is not needed though can be requested.

Alternatively, electronic consent may have benefits when used in a non-remote fashion. For example, the trial or study may still be run in a clinic or hospital setting, but eligible patients may present out of hours when researchers are not working. In this case, having an electronic consent on hand to recruit them in the first instance can be valuable [4] by potentially cutting down on the amount of time required of research personnel. This use of electronic methods to conduct all or part of the trial recruitment process (from invitation through screening, informing, to obtaining informed consent) is known as electronic consent or e-consent ([12], Introduction p40).

### Uses of e-consent in CTIMPs to date

The use of e-consent in CTIMPs to date is uncommon. Although there are published studies with RCT designs which use e-consent, the majority of these are not the consent of real participants to real CTIMPs. Instead, they test e-consent processes in ‘mock’ or ‘simulated patient’ situations where a hypothetical trial is proposed but the e-consent testers are not in an active study or trial [4, 13] Other studies which used e-consent were neither RCTs nor interventional, following observational or health data sharing designs [14]. In these studies, some e-consent processes were tested for user satisfaction without a comparator, and at other times were compared with paper formats [15, 16].

A few CTIMPs have used an e-consent process on real trial participants [8, 10, 17–19]. Of these, many were low risk as they used licensed drugs [8, 10, 19]. Haussen et al. [17, 18] used e-consent in interventional stroke trials, one of a drug, and the others of devices for thrombectomy. Here the context of using e-consent was more ethically complex as risks were more than minimal, and they used legally authorised representatives (LARs) to provide consent on the patients’ behalf. Their evaluations of the e-consent process found efficiency savings from e-consent and a statistically significant user preference for e-consent over paper [18]. Haussen [17] found that the time from door-to-randomisation improved using e-consent. Evidence to date, therefore, is fairly limited, but indicates modest support for using e-consent in CTIMPs on practical grounds, and clearly requires further study. In all of this, however, the ethical grounds for using e-consent in CTIMPs are far less clear.

### Understanding ethical reasons for e-consent as specifically applied to use in CTIMPs

The literature on the ethics of e-consent in CTIMPs is underdeveloped. This is largely because certain features of using e-consent in CTIMPs which are ethically significant have been overlooked or over-emphasised at the expense of other key considerations. Here, we describe four of these features and show that there are a broader range of ethical considerations to be understood in the ethics of e-consent.

#### E-consent in ‘mock’ trials can, at best, provide only a limited picture of the ethics landscape

A number of issues arise regarding the empirical data surrounding e-consent. First, this data, which is taken to inform e-consent’s acceptability, largely comes from mock tests of e-consent on simulated participants. This risks providing only a limited picture of the ethical considerations relevant to e-consent. This is not to say that mock e-consent studies have not generated some useful starting considerations. These studies report benefits from using e-consent including logistical efficiencies [17] (also for efficiency given pandemic conditions, see e.g. [20] NeuroSAFE and [19] PRINCIPLE), increased comprehension of information [21], ability to test comprehension [14], ability to present information in more meaningful ways e.g. tiering [22] and better user experience [18]. One frequently stated risk is that e-consent might increase the social inequality of access to trials [5, 20], also referred to as increasing the ‘digital divide’ between technologically savvy and less savvy groups, for which there is modest empirical support [23, 24]. Other studies noted a further risk that ‘cultural conditioning’ which causes us to agree to other types of e-consent without scrutiny (e.g. in click through agreements) might lead to poorly informed or otherwise illegitimate e-consent in medical research [22, 25]. To mitigate the mindless click through effect, Wilbanks suggests that ‘cognitive friction’ — a phenomenon which refers to glitches in design communication processes causing users to stumble over content — should be deliberately built into e-consent processes to encourage users to ask questions, express doubt and self-reflect on trial participation.^5^

These starting considerations are interesting but provide a limited picture of the ethics landscape for the following reasons. First, mock e-consent users have a lack of ‘skin in the game’: they are not genuinely presented with terms of participation and are not faced with the prospect of undergoing risk in any real sense. This means we have few empirical insights into how real trial participants understand risk in an e-consent process, nor how they deal with other common CTIMP comprehension pitfalls such as understanding of placebo controls and randomisation. Because the consent is broadly hypothetical, how participants actually understand these processes matters less (or perhaps differently) both for them and for the ethical acceptability of the consent. Second, we lack insight into the experiences of researchers and trial staff who are administering electronic consent processes in reality, including how these processes might combine with real phone and video interactions with patients who have questions. This misses interesting considerations about whether e-consent depends on human interaction for its administration, as well as questions about how reactive and flexible a consent process should be in response to particular participants’ needs.

The second set of issues arising from this, albeit limited, empirical data on e-consent is its actual relationship to an account of ethical acceptability. The broadest form of this set of issues points to the distinction between what is ethically acceptable and what is taken to be ethically acceptable. Famously, it is often supposed that if we asked people generally whether they thought capital punishment was an acceptable form of punishment, the comfortable majority would say that it is. However, we would rightly be hesitant to take it to follow that capital punishment is ethically acceptable. This kind of thought experiment shows that ‘what is ethically acceptable’ and ‘what is taken to be ethically acceptable’ can come apart in important ways. In the context here, this means that we should be careful of how we extrapolate from empirical findings which show approval in one form or another to ethical acceptability: we cannot simply read ethical acceptability off from participant approval.

In concrete methodological terms, this gap can be bridged by combining or integrating empirical approaches with conceptual, normative methods. So rather than simply asking participants for their opinions of the process, the researcher should ask for the reasons or justifications of those opinions and could potentially engage the participant in reflection on the adequacy of them. These methods are well described in the literature on empirical ethics methodologies [27, 28]. They have the advantage of getting at the reasoning processes behind an avowed approval and allowing it to be scrutinised in terms of argumentative content and force. As researchers we are then in a position to know, not just what stakeholders think, but why they do so.

Overall, then, to fully understand the nature and ethical ramifications of e-consent, it should be used in real CTIMPs by real participants and other stakeholders, then analysed using a mix of empirical and conceptual, normative approaches.

#### E-consent is not (just) documenting consent

A second feature of current discussions of e-consent that is important here is an over-emphasis on the documentation of e-consent rather than on the e-consent processes themselves. This is found in some of the e-consent literature and regulations which seem overly focused on documentation-related questions, such as what standards are appropriate in order to verify a person’s identity when they sign an electronic consent form, and how to ensure personal information is transferred and stored securely [12]. For example, the UK joint statement seems very much dominated by the verification question (see Summary p1). These concerns are ethically relevant to an extent — we should certainly protect participants’ safety and confidentiality by having robust systems in place to verify their identity, and trial datasets should not be corrupted by rogue data. However, these concerns are also fairly uncontroversial, ethically speaking, and clearly can be managed by legal and regulatory safeguards.

The distinction between consent and its documentation is worthy of comment. In the normal course of events, we want to ensure that the potential participant actually makes a decision to participate. This is distinct from ensuring that it is actually the participant who makes the decision. It is the decision which matters. But when we focus on guarantees about the identity of the participant, the focus shifts from the decision to the decision-maker. The identity of the decision-maker matters ethically when having someone else decide has direct consequences for the person being ‘decided for.’ In the case of remote consent or e-consent, it is hard to see what the direct, pressing consequences could be. If the wrong person takes the medication this is of concern, but this is not resolved by ensuring identity — and we can reasonably assume that people generally do not take unknown medication. There may be concerns about access to data without proper permission, but the risk of harm here seems quite remote and certainly not blameworthy. In general, these concerns seem to be mostly borne out of a misplaced worry about the lack of control outside of the clinical setting. But of course, the same autonomy and the same ability to make independent responsible choices, both of which are to be respected in the clinical setting, is present in the home.

What needs more recognition is that e-consent in CTIMPs may encapsulate the whole recruitment process from invitation through to documenting consent. This might involve using electronic invitation, screening stages, information provision and interactive discussion (video/phone calls with real researchers or potentially a live chat system) during the participants’ digest of the information and comprehension stages. This is then followed by electronic documention of consent.

Documenting consent is not consent. Although the final signing of a document can represent the act of making a (final) decision, the signing is the contingent marking of that decision and could just as easily be captured in multiple alternative ways [3]. The earlier parts of the process, those that crucially contribute to the decision-making process, raise many more ethical questions to do with comprehension and voluntariness, as well as the role of relational factors e.g. the role of participant trust in an electronic consent process versus one delivered by humans.

#### E-consent should be distinguished from dynamic consent

Third, it is important to emphasise that e-consent is not dynamic consent and specifically could not mean dynamic consent in the CTIMP case. This is because dynamic consent is consent iterated over time (albeit often electronically) to multiple research opportunities, allowing participants to opt in and out of multiple studies [29]. E-consent to a CTIMP, however, is (and must be) a specific consent to a particular interventional protocol. This view conflicts with some of the literature on e-consent which either implicitly or explicitly characterises it and its benefits/risks as those of dynamic consent. For example, Petrini et al. warn that a risk of decentralised trials using e-consent is that consent could go ‘beyond the study scope’, so indicating a conflation between dynamic consent and e-consent. Also, de Sutter et al. define electronic IC as ‘an interactive online-based IC application which could facilitate interactions over time and could enable a personalized approach, adapted to research participants’ needs’ [30]. They also note a benefit of e-consent as being the ability to give feedback and return results to participants, even tailoring them to be relevant to participants’ particular interests or clinical features. In both of these cases, e-consent is confused with the use of an ‘electronic’ platform or system: clearly e-consent requires some form of electronic platform, but it does not entail all uses of such platforms.

While a personalised approach to feedback and results might be useful functions of a trial data management system, they do not relate to e-consent in particular, nor are they relevant arguments for (or against) using it in a CTIMP. This is because if we equate the benefits of e-consent too closely with tailoring of information, we may over-associate e-consent with the ability to frame or withhold information. This involves distinguishing the presentation of information from personalising information. The trial consent literature has advocated improvements to how information during IC is presented, such as using ‘choice architecture’ or tiered consent models such that information is more digestible to different educational and cultural backgrounds [22, 30, 31]. E-consent platforms lend themselves to this tiering approach, alongside other approaches such as the use of visuals and animation to replace dry written information.

This is different from tailoring or personalising information, which relies on the designer of the IC process anticipating or deciding how much information to give to a particular participant or cohort, potentially withholding some information. This makes assumptions about what people want to know and how much information is sufficient to make an informed decision, with substantial ethical and legal risks [32]. Therefore, one important consideration is being clear that we should not choose for participants the information we think they would want to know during e-consent (though we might more defensibly do this at later ‘feedback’ stages of trial participation). Nor should we imply — as the conflation of consent with dynamic consent discourse invites — that certain participants should be excluded from being offered at least essential trial information during a CTIMP e-consent process, even though they may choose not to receive the offer. This is granting that issues of information overload and comprehension still need managing in any appropriate e-consent process. So, in order to be clear about what e-consent to CTIMPs entails, we should distinguish it from the dynamic consent model.

#### The ethical importance of ‘in-person’ meetings and ‘two-way communication’ in consent

The final under-considered set of issues involves the ethical significance of ‘in-person’ or ‘two-way communication’ interactions during consent processes generally. Clearly, if these features matter, then work is needed on appropriately preserving or replicating them in the e-consent context. Importantly, some literature and some regulatory guidance on e-consent have stressed the importance of maintaining the option for in-person interaction and ‘two-way communication’ during the consent process [5, 12, 33], perhaps on the basis that this would be part of conventional consent, but these examples lack sustained critical attention.

Notably, the UK regulator’s guidance prefers an in-person interview to form part of the e-consent process, unless this is not possible [12]. When it is not possible, preferably there should still be a ‘two-way communication in real-time’ by telephone, video conferencing or similar. This is both in order to verify identity and because such a communication delivers the interactivity of an in-person interview, which ‘allows the participants to ask questions and receive answers from the investigator or member of the investigating team’ (2018 p6).

However, it may be that the need to preserve in-person interaction is overstated. In particular, if we are less concerned about the verification of identity issue, then it may be that the in-person connection is of less significance for understanding than it may first appear. It is unclear what extra ethical value meeting in-person gives us. On the face of it, there are clear methods for answering questions which do not require another person, either on a call or in-person.

The answer to this question partly turns on demonstrating the ethical value of in-person interaction in the conventional consent setting, perhaps that it welcomes the asking of questions or engaging with the trial material in a way that enhances consent. Then a further claim would need to be made about whether e-consent discourages questions and engagement. However, this makes assumptions about both the conventional and electronic consent context. In a conventional context, there may be no meaningful meeting or communication. Instead, being handed a paper Patient Information Sheet (PIS) and consent form may be all that time and resources allow for. Equally, the electronic context may provide better engagement with material, more time and allow for questions to be asked and answered at times convenient to participant and researcher. Additionally, interacting in-person may have disadvantages associated with biases and assumed relationships, such as heightening the ‘therapeutic misconception’ [34] or leading to an over-reliance on assumptions about roles and relational trust between the clinician and the patient at the expense of comprehension and clearly focussed choice [35]. For similar reasons, we should query the preference for ‘two-way’ or ‘thorough and interactive’ communication that goes over and above what is technologically available. In some cases, thorough, interactive interpersonal communication may be useful in order to engage patients and answer their queries, but in others, it may introduce unnecessary resource costs. In the context of modern technology, with automated chat facilities and the ability to clarify and answer questions electronically, the question remains as to what extra role and extra value is provided by live, person-to-person interaction.

The issue of in-person and two-way interaction is therefore important to subject to further ethical analysis but not perhaps for the reasons given by the literature and guidance so far. In fact, the issue points to ways in which conventional consent may not be all as it seems — in theory, conventional consent facilitates an informed, voluntary agreement but in practice may be more reliant on trust with the investigator and subtle power dynamics, which work against what is ethically important about the process. One interesting question arising from this is whether such reliance poses a kind of ethical ‘problem’ for the legitimacy of consent, and therefore whether adopting e-consent would or should correct the problem (for example, of therapeutic misconception, or overly trusting relationships).

### Towards a better understanding of the ethics of e-consent in CTIMPs

In the above, we have described some challenges to using conventional consent processes in large-scale clinical trials in population health. Alternatives are being developed which might better suit the designs of these trials and other remote or decentralised trial designs. These alternatives include electronic consent (e-consent) processes spanning from invitation through screening, information-giving, and consent stages. To date, the use of e-consent processes in real CTIMPs remains uncommon but indicates (alongside mock studies of e-consent) good practical reasons for its use. However, the ethical grounds for using e-consent in CTIMPs need further attention and research.

We have sketched four features of the broader ethical landscape connected to the use of e-consent in CTIMPs which further focused on ethical analysis. Importantly, developing ethical grounds for using e-consent should avoid reproducing existing ethical problems associated with conventional consent processes. This involves giving a proper account of what consent should accomplish in contemporary clinical trials when designing future e-consent processes.

We have argued this work should begin by addressing some features of e-consent which have been overlooked or contrastingly over-emphasised in terms of their ethical significance. Firstly, we should use e-consent in real CTIMPs and evaluate its effectiveness and ethical acceptability. Secondly, we should consider ethical questions arising throughout the e-consent process overall, not just at the digital consent or identity verification stage. Thirdly, we should separate the analysis of e-consent from the discourse on dynamic consent, in order to be clear about the risks of over-framing or personalising information during consent. Finally, we should question the extent to which preserving features of conventional consent such as in-person interaction and two-way communication in e-consent processes is required, for fear of reproducing the problems of consent in the digital age.

## Data Availability

Not applicable.

## Footnotes

1 Where some clinic visits are still needed, this is termed decentralised or hybrid clinical trials [5, 7].

2 https://www.ascend-plus-trial.org/.

3 https://www.ctsu.ox.ac.uk/lens.

4 Note though e-consent is most logically suited for remote designs, we have found at least one RCT (TOPAZ) where e-consent is used even though research nurses physically interact with the participants (in order to infuse the study drug) and so could conduct some parts of the consent process with participants in-person.

5 Interestingly, this makes important presumptions about the ethical inadequacy of ‘click through’ consent in the context of CTIMPS: namely, that competent individuals are incapable of deciding whether this example of ‘click through’ consent involving a CTIMP is distinct from the ‘click through’ consent on another kind of webpage [26].
